# Associations between C-reactive protein levels and cognition during the first 6 months after acute psychosis

**DOI:** 10.1017/neu.2018.25

**Published:** 2018-11-05

**Authors:** Farivar Fathian, Else-Marie Løberg, Rolf Gjestad, Vidar M. Steen, Rune A. Kroken, Hugo A. Jørgensen, Erik Johnsen

**Affiliations:** 1 NKS Olaviken Gerontopsychiatric Hospital, Bergen, Norway; 2 Department of Addiction Medicine, Haukeland University Hospital, Bergen, Norway; 3 Department of Clinical Psychology, University of Bergen, Bergen, Norway; 4 Division of Psychiatry, Haukeland University Hospital, Bergen, Norway; 5 Department of Clinical Science, NORMENT and KG Jebsen Centre for Psychosis Research, University of Bergen, Bergen, Norway; 6 Dr. Einar Martens Research Group for Biological Psychiatry, Center for Medical Genetics and Molecular Medicine, Haukeland University Hospital, Bergen, Norway; 7 Department of Clinical Medicine, Section Psychiatry, University of Bergen, Bergen, Norway

**Keywords:** C-reactive protein, cognition, inflammation, psychosis, schizophrenia

## Abstract

**Objective:**

Inverse relationships between the C-reactive protein (CRP) levels and cognitive performance in acute psychosis have been demonstrated. We aimed to investigate how the serum level and initial change of CRP in acutely admitted patients with psychosis was correlated with cognitive performance during a 6-months follow-up period.

**Methods:**

The study is part of a pragmatic, randomised trial comparing four different second-generation antipsychotic drugs, and consists of 208 acute phase patients recruited at admittance for psychosis. This study reports data for all groups collectively, and does not compare treatment groups. Measurements of CRP and cognitive performance were conducted at baseline (T1) and after 4 weeks on average after inclusion (T2). Cognition was also assessed after 3 months (T3) and 6 months (T4) of follow-up.

**Results:**

Global cognition improved during the follow-up period of 6 months, especially in the T1–T2 interval. The different cognitive subdomains showed different time-dependent profiles of improvement, with memory and attention improving significantly also in the later phases. Reduction of the CRP level during the initial follow-up interval (T1–T2) was associated with increased overall cognitive performance in the T2–T4 interval, but not in the T1–T2 interval. For the cognitive subdomains, we found an inverse association between change in CRP level and verbal abilities (T2–T4 interval), and attention (T2–T3 interval).

**Conclusion:**

These findings indicate that initial changes in the serum level of CRP in the acute phase of psychosis may predict cognitive function in later phases of the disease.

## Trial registration

ClinicalTrials.gov ID; URL: http://www.clinicaltrials.gov/:NCT00932529

**Significant outcomes**

∙ Global cognition and several cognitive subdomains continued to improve beyond the initial phase of acute psychosis treatment and into the later phase of follow-up.
∙ Reduction of the C-reactive protein (CRP) level in the initial acute phase was associated with delayed improvement of global cognition. Inverse associations between CRP levels and cognitive subdomains were found for learning, verbal abilities, and attention.

**Limitations**

∙ There was considerable attrition in the study during follow-up.
∙ CRP was the only inflammatory marker measured in the study.

## Introduction

Cognitive dysfunctions are core features of schizophrenia that detrimentally affect functional outcome (1–3). The neurobiological disturbances involved in cognitive dysfunctions are, however, largely unknown. Abnormal myelination, white matter changes, and immunological processes, including low-grade inflammatory responses, have been suggested as possible mechanisms (4). Many studies have indicated that the cognitive impairments seem to stabilise after the first episode of psychosis (5,6), although there have also been some findings suggesting cognitive improvements (7–10). The inconsistent literature might at least in part be explained by differences in the patient samples, the duration of follow-up, and the cognitive test batteries. Taken together, recent findings indicate that cognitive dysfunction is not always irreversible and can be dynamic, especially in the acute phases (11,12). Importantly, current treatment options in psychosis, including psychosocial interventions and antipsychotic medications, have few if any beneficial effects on cognitive performance (4,13–17). Thus, this central area of dysfunction in psychosis is in need of a better understanding and novel treatment approaches.

Inflammation has been the focus of recent studies addressing the pathophysiology of psychotic disorders. Immune abnormalities in the blood, cerebrospinal fluid, and central nervous system, including immune cell numbers, inflammatory markers, and antibody titers, have been demonstrated in schizophrenia (18–23). There is increasing evidence for underlying inflammatory mechanisms relevant to cognitive functioning (4,24–26). Indeed, neuroinflammation with white matter pathology has been demonstrated in the early stages of psychosis (27), and it has been suggested that these changes can lead to both structural and functional dysconnectivity and cognitive dysfunction (21). Low-grade elevation of the CRP, a well characterised and standardised marker of inflammation (28), has been found in some studies in schizophrenia (29–35). An inverse association between the serum levels of CRP and cognitive function has been reported (18,36,37). However, these studies are predominantly cross-sectional and include patients in the chronic phase. This might restrict the generalisability of the results, and longitudinal studies with repeated cognitive assessments are scarce.

We have recently shown an inverse association between the serum level of CRP and cognitive performance in the acute phase of psychosis (11). What remains unresolved, however, is whether the levels and change of CRP in the initial phase of acute psychosis influence cognition in later phases. The main aim of this study was therefore to investigate how the CRP level in the first acute phase of psychosis correlates with cognitive function during a 6 months follow-up.

## Material and methods

**Figure 1:**
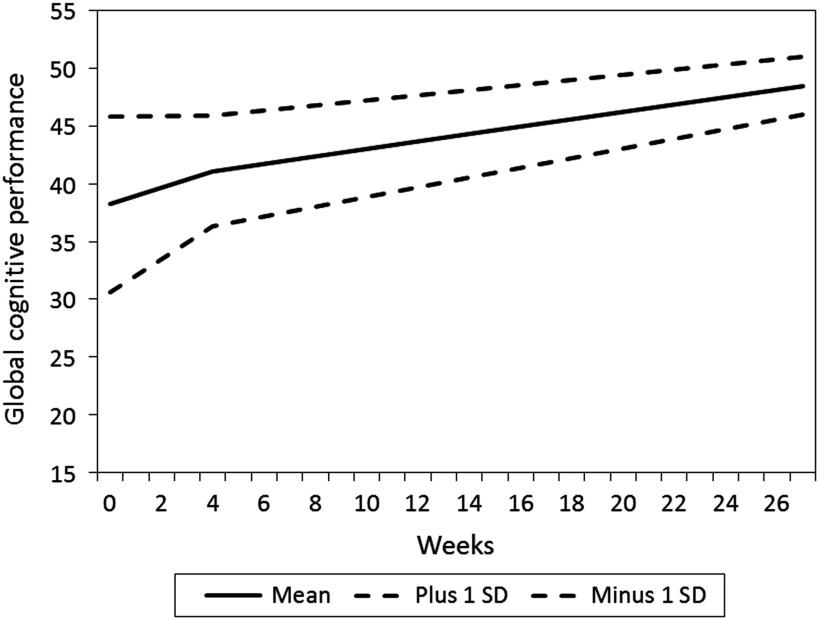
Estimated mean level and change in global cognitive score over time, with standard deviations to display individual variation (*N*=181).

The study is part of a pragmatic, randomised trial comparing four different second-generation antipsychotics in the treatment of psychosis (Fig. 1). Importantly this study reports data for all treatment groups collectively, and does not compare treatment groups. We have previously published cross-sectional comparisons of treatment groups, and no differences were found (11). The main objective of the present study was to investigate overall associations between longitudinal changes in CRP and cognition in the whole group. The clinical sample and methods have been described in detail elsewhere (38).

### Clinical sample

The sample consists of 208 consecutive, acute phase patients recruited at admittance for psychosis, who underwent CRP measurements and cognitive assessment. The project was designed and approved by the Ethics committee with two phases: The first was the quality assurance phase from admission to discharge or 6 weeks at the latest, and was approved by the Ethics Committee without the requirement of informed consent as this phase included only elements of best clinical practice. At admission all psychotic patients admitted to the hospital for symptoms of acute psychosis were consecutively included, if they were to use the hospital’s standard antipsychotic medication regimen, and could cooperate with clinical assessments of condition. This phase should assure that psychosis patients were offered best-quality guideline-concordant treatment for psychosis. The second phase (research phase) was based on informed consent provided at discharge or after 6 weeks at the latest. This included invitation to visits and tests at 3, 6, 12, and 24 months after admission. In this part of the project there were procedures beyond usual clinical standard, such as collections of data for use in psychiatric basic research within genetics and brain functioning.

The recruitment period was from March 2004 until February 2009 at Haukeland University Hospital, Bergen, Norway, with a catchment population of about 400 000. The study was approved by the Regional Committee for Medical Research Ethics, and by the Norwegian Social Science Data Services. The study was publicly funded and did not receive any financial or other support from the pharmaceutical industry.

All adult patients were eligible for participation if they were acutely admitted to the emergency ward for symptoms of active psychosis, as determined by a score of ≥4 on one or more on the items Delusions, Hallucinatory behaviour, Grandiosity, Suspiciousness/persecution, or Unusual thought content in the Positive and Negative Syndrome Scale (PANSS) (39) and were candidates for oral antipsychotic drug therapy. All eligible patients met the International Statistical Classification of Diseases and Related Health Problems (ICD)-10 diagnostic criteria (http://apps.who.int/classifications/icd10/browse/2010/en) for schizophrenia, schizoaffective disorder, acute and transient psychotic disorder, delusional disorder, drug-induced psychosis, major depressive disorder with psychotic features, bipolar disorder except manic psychosis. Manic psychosis was excluded based on *a priori* expectation that these patients would not be able to cooperate with the assessments in the acute phase.

Patients were excluded from the study if they were unable to use oral antipsychotics, were suffering from manic psychosis or, for other behavioural or mental reasons related to the state of illness, were unable to cooperate with assessments, did not understand spoken Norwegian, were candidates for electroconvulsive therapy, or were medicated with clozapine on admittance. Patients with drug-induced psychoses were included only when the condition did not resolve within a few days and when antipsychotic drug therapy was indicated.

The patients were assessed at baseline (T1: *N*=208 with data on CRP level and/or cognition), at the first follow-up (on average after 4 weeks; T2: *N*=103), at the second follow-up after 3 months (T3: *N*=43), and at the third follow-up after 6 months (T4: *N*=35). This period corresponds to the treatment of the acute phase during which the largest reduction of psychotic symptoms is seen, and treatment response is expected in the majority of patients (40).

### Clinical and biochemical assessments

Eligible patients underwent the PANSS structured clinical interview at baseline, before inclusion. All tests were performed by personnel independent of the clinical departments, who were trained and with inter-rater reliability training. Intra-class correlation coefficients were calculated based on inter-rater assessments and showed high inter-rater reliability (0.92). Furthermore, the Calgary Depression Scale for Schizophrenia (41), and the Clinical Drug and Alcohol Use Scales (42) were used, and the patients were rated according to the Clinical Global Impression-Severity of Illness Scale (43), and the Global Assessment of Functioning-Split Version, Functions Scale (44). Illicit drug use data was based only on information from the participant.

For analysis of CRP, a blood sample was collected from the patients in the fasting state between 08:00 and 10:00 h in the morning at baseline (T1) and after 4 weeks follow-up (T2). The laboratory changed the routine CRP analysis method in January 2005, and hence only data obtained after this revision is used in the present work. The CRP level was measured by the Tina-quant C-reactive Protein (Latex) method from Roche Modular P^®^ (Mannheim, Germany), which measures CRP levels >1 mg/l.

### Cognitive assessment

Cognitive assessments were conducted at baseline (T1) and follow-up (T2, T3, and T4). A brief neuropsychological screening instrument with alternative forms; the Repeatable Battery for the Assessment of Neuropsychological Status (RBANS), was used to minimise potential practice effects (45–47), since longitudinal studies on cognitive functioning usually do not sufficiently address the issue of practice effects (48,49). The RBANS has previously shown to have good reliability and validity in psychosis (47). To increase clinical validity, a more comprehensive cognitive battery, but with the same cognitive subdomains, was administered at T3 (Table 1). In a preliminary analysis, a satisfactory relationship between the RBANS/short battery and equivalent domains from the long battery/more comprehensive neuropsychological battery 3 months later emerged, even despite of symptom-level differences at the two time points (50). The five cognitive domains were the same as for the RBANS: Language; Visuospatial/constructional; Immediate memory; Delayed memory; and Attention. We have used the already set domains from the RBANS, which does not include executive functioning. We do argue, however, that both Trails B and Stroop do depend as much on attentional resources, as executive functioning making it correct to group them together with the Attention domain. If we had an executive domain we would have grouped in this domain also. The policy of grouping test with several domains when they do capture several cognitive processes is what we consider the most transparent way of taking the complexity into account. The grouping of WAIS III digit span with both Attention and Learning was done for the same reason. In addition, in the before mentioned preliminary analysis, this method of grouping the tests was the one that obtained a satisfactory relationship with the RBANS (50). Raw scores for the neuropsychological variables were converted to *t*-scores using the best available norms from corresponding manuals (when available) or published papers. The final summary score based on the mean *t*-scores across the five cognitive domains defined the overall cognitive function *t*-score. Neuropsychological assessment by cognitive domain and time are shown in Table 1.

**Table 1:** Neuropsychological assessment by cognitive subdomain and time

Cognitive subdomain	T1 (baseline)	T2 (4 weeks*)	T3 (3 months)	T4 (6 months)
Verbal abilities	RBANS A language	RBANS B language	WAIS-III similarities WAIS-III vocabulary COWAT letters/animals	COWAT letters/animals
Visuospatial abilities	RBANS A visuospatial/constructional	RBANS B visuospatial/constructional	WAIS-III digit symbol-coding WAIS-III block design ROCF copy	ROCF copy
Attention	RBANS A attention	RBANS B attention	WAIS-III digit span Digit vigilance test (time)CalCAP CPT (choice reaction time/sequential reaction time) Trail making test B Stroop colour/word conflict (time)	WAIS-III digit span CalCAP CPT (choice reaction time/sequential reaction time) Stroop colour/word conflict (time)
Learning	RBANS A immediate memory	RBANS B immediate memory	CVLT-II (immediate recall) WMS-R logical memory WAIS-III digit span	WMS-R logical memory WAIS-III digit span
Memory	RBANS A delayed memory	RBANS B delayed memory	CVLT-II (delayed recall) ROCF delayed recall	ROCF delayed recall

CalCAP CPT, California Computerized Assessment Package-Continuous Performance Test; COWAT, Controlled Oral Word Association Test; CVLT, California Verbal Learning Test; RBANS, Repeatable Battery for the Assessment of Neuropsychological Status; ROCF, Rey Osterrieth Complex Figure Test; WAIS-III, Wechsler Adult Intelligence Scale; WMS-R, Wechsler Memory Scale-Revised.

* Mean follow-up time at T2 was 4.1 weeks after baseline.

### Statistical analyses

Descriptive statistics (mean, standard deviation, frequency) and independent sample-*t*-test were analysed with SPSS version 24.0. The program Mplus 8 (51) was used to analyse the level and change in variables with latent growth curve and time contrast models (52). The standard linear change assumption was tested based on the goodness of fit measures, residual results, and plots of the observed and estimated change model were used to decide the change pattern. If this assumption was not met, piecewise growth was explored. If the model indicated one piece based on two measurements only, this part is a difference score model (53). Some residuals had to be set as constant to fit such a model. The models were evaluated based on their goodness of fit measures, as follows: threshold values in comparative fit index and Tucker–Lewis index beyond 0.95; root mean error of approximation (RMSEA)<0.05 as close fit, RMSEA <0.08 as fair fit, and RMSEA <0.10 as a mediocre fit (52). Model fit measures were presented for unconditional level and change models, but not for prediction models, as these models solely are predictions with statistical significant and non-significant relations and not structural models. The estimator was set to maximum likelihood with robust standard errors (MLR) to account for non-normality in data (54). The full information maximisation likelihood (FIML) method uses all available data under the assumption that missing data are random (‘Missing at Random’) (51,54). Thus, the level and change results are estimated and not observed values based on all observations. After analysing the variables separately, level and change in CRP were related to levels and changes in neurocognitive scales. A sensitivity analysis was performed for CRP levels <10 mg/l.

Finally, we controlled for confounders, known to have impact on both CRP level and cognition, as metabolic syndrome, smoking, being antipsychotic medication-naive, illicit drug use, and educational level (11).

## Results

The study included 208 patients with data in one or more of the outcome variables. At baseline, there were CRP measurement for 158 patients and cognitive assessments for 169 patients and a total of 123 patients had both measurements. In total, 181 patients had observations on cognitive performance and 169 on CRP at any time point. The demographic and clinical characteristics are shown in Table 2. The patients that were tested only at baseline were not statistically different from those with follow-up data for the clinical or demographic characteristics at baseline, with the exception of a higher PANSS negative subscale score in patients with baseline test only compared to those with two or more visits [independent samples *t*-test: *p*=0.034; mean difference 2.2 points; 95% confidence interval (CI): 0.2–4.2], and fewer years of education (independent samples *t*-test: *p*=0.027; mean difference, 0.8 years; 95% CI: 0.10–0.6).

**Table 2:** Baseline demographics and clinical characteristics (*N*=208)

Characteristics	*N*	%	
Male	143	68.8	
Antipsychotic drug naive	92	44.2	
Alcohol abuse last 6 months	22	10.6	
Illicit drug use last 6 months	139	66.8	
Current tobacco smoking	103	49.5	
Diagnosis*			
Schiz and related	106	53.2	
Drug-induced	28	14.1	
Affective	23	11.6	
Acute psychosis	17	8.5	
Other	25	12.5	
	Mean	SD	Range
Age	33.5	13.1	17–73
Body mass index	23.7	4.8	15.8–42.6
Years of education	12.5	2.8	8.0–22.0
PANSS total	74.1	13.2	44–111
PANSS positive	19.9	4.4	11–32
PANSS negative	19.6	7.4	7–39
PANSS general	34.6	6.7	20–56
CDSS	6.6	5.2	0–23
GAF-F	30.7	6.0	2–62
CGI	5.2	0.6	4–6
RBANS	38.3	7.7	20.2–58.8
CRP	4.0	8.3	0–89

*N*, number of patients; Antipsychotic drug naive, no life-time exposure to antipsychotic drugs before index admission; Abuse, abuse or dependence according to the Clinical Drug and Alcohol Use Scales (CDUS/CAUS), patients with no illicit drug use could be included in the category alcohol use last 6 months; Schz and related, schizophrenia and related disorders: Schizophrenia, schizoaffective disorder, acute polymorphic psychotic disorder with symptoms of schizophrenia, acute schizophrenia-like psychotic disorder, delusional disorder; Acute psychosis, acute psychosis other than those categorised under Schz and related; Affective, bipolar and unipolar depression; Other, miscellaneous psychotic disorders. CDSS, the Calgary Depression Scale for Schizophrenia; CGI, the Clinical Global Impression, severity of illness scale; CRP, C-reactive protein (mg/l); GAF-F, the Global Assessment of Functioning, split version, Functions scale; PANSS, the Positive and Negative Syndrome Scale; RBANS, the Repeatable Battery for the Assessment of Neuropsychological Status. All diagnoses are according to ICD-10;

* Patients with missing diagnoses are not included in the list. According to the naturalistic design, patients were included on the basis of the presence of active psychosis as determined by the PANSS, and not based on diagnosis.

### Levels and changes in cognitive performance

The mean and individual levels and changes in cognitive subdomains are presented in Table 3. The mean level of global cognitive performance increased over time in a non-linear manner, being most pronounced in the T1–T2 interval (Fig. 1). The figure also shows more individual change variation in the T1–T2 interval than in the later intervals. Statistically significant improvements over time were also found for verbal abilities, learning and attention in the T1–T2 interval, and for memory and attention in the T2–T4 interval, respectively. Individual differences in change (standard deviation) were also present. Some patients improved their performance, whereas others showed decline. The goodness of fit measures showed close fit between model and data for global cognitive performance, verbal abilities and learning scales, while the other three scales were based on saturated models and thereby giving no fit measures (please see Supplementary Table 1).

**Table 3:** Levels and changes in global cognition and cognitive subdomains over time

	T1 Level (I)			Change T1–T2 (S1)				Change T2–T4 or change T2–T3 (S2)*				Change T3–T4 (S3)*			
Scale	Mean	SD	*p*	Mean	*p*	SD	*p*	Mean	*p*	SD	*p*	Mean	*p*	SD	*p*
Global performance	38.3	7.6	0.000	2.9	0.000	4.9	0.000	1.4	0.000	1.1	0.003				
Verbal abilities	40.1	8.4	0.000	6.0	0.000	6.8	0.000	0.2	0.643	2.0	0.002				
Visuospatial abilities*	47.3	12.1	0.000	1.4	0.148	10.1	0.000	−0.7	0.295	5.1	0.000	2.1	0.000	3.2	.004
Learning	35.8	8.1	0.000	2.9	0.000	1.7	0.078	−0.3	0.315	1.9	0.000				
Memory*	38.1	12.1	0.000	1.2	0.326	11.3	0.000	2.8	.000	4.8	.000	3.0	.000	2.3	.000
Attention*	29.7	9.0	0.000	3.5	0.000	7.4	0.000	4.2	.000	4.0	.000	0.4	.167	2.1	.000

The time unit for change is months. T1, baseline; T2, 4 weeks; T3, 3 months; T4, 6 months. S1: change from T1–T2; S2: change from T2–T4 if supported by data; and S3: change T3–T4. Change in the T3–T4 interval was estimated if a linear change from T2–T4 was not supported. In these models, S2 consists of the change between T2–T3. Group mean and individual differences (standard deviation, SD) for both level and change are reported. Statistical *p*-values are presented for individual differences at baseline level (T1 Level), and mean change and individual differences in change (standard deviation).

* Three change factors had to be estimated: S1: T1–T2; S2: T2–T3; and S3: T3–T4.

### The relationship between levels and changes in CRP and cognition

**Figure 2:**
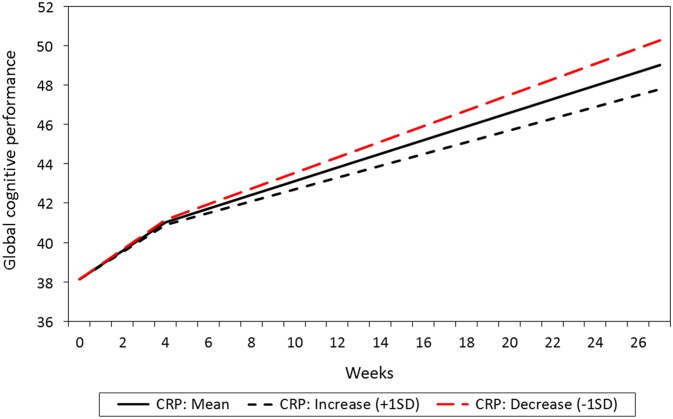
Relationship between estimated level and change in C-reactive protein (CRP) and global cognitive performance total score (*N*=208).

The mean change in CRP from T1 to T2 was not statistically significant (0.52 mg/l/ month, *p*=0.652, baseline mean level: 3.99 mg/l, SD =8.32). However, the individual variation in change was statistically significant (SD =12.13, *p*=0.031). In the total sample, a reduction in the CRP level from T1 to T2 was associated with an increase in global cognitive performance in the T2–T4 interval, whereas no such association was found between CRP and cognition during the T1–T2 interval (Table 4). Patients with most reduction in CRP also were the patients with most improvement in global cognitive performance, illustrated with a steeper increase in their performance, compared to those with a smaller reduction or an increase in CRP (Fig. 2).

**Table 4:** Baseline level and changes over time in cognitive performance predicted by baseline and change in C-reactive protein (CRP) level

	Total sample						Subsample (CRP <10 mg/l)					
	CRP level T1			CRP change T1–T2			CRP level T1			CRP change T1–T2		
	*b*	β	*p*	*b*	β	*p*	*b*	Β	*p*	*b*	β	*p*
Global performance												
T1	−0.35	−0.37	0.002	–	–	–	−0.70	−0.17	0.059	–	–	–
T1–T2	0.13	0.21	0.455	−0.01	−0.03	0.748	0.95	0.34	0.013	−0.02	−0.04	0.573
T2–T4	0.08	0.54	0.025	−0.02	−0.18	0.004	−0.07	−0.12	0.684	−0.02	−0.14	0.026
Verbal abilities												
T1	−0.35	−0.35	0.120	–	–	–	−0.75	−0.17	0.095	–	–	–
T1–T2	0.13	0.17	0.610	−0.06	−0.10	0.124	1.11	0.30	0.082	−0.07	−0.09	0.103
T2–T4	0.11	0.39	0.197	−0.04	−0.23	0.003	0.17	0.15	0.600	−0.03	−0.14	0.039
Visuospatial abilities												
T1	−0.28	−0.19	0.067	–	–	–	−0.49	−0.07	0.434	–	–	–
T1–T2	0.56	0.44	0.059	0.04	0.05	0.724	0.94	0.16	0.146	0.05	0.05	0.684
T2–T3	−0.31	−0.48	0.111	−0.05	−0.11	0.416	−0.96	−0.34	0.048	−0.06	−0.11	0.311
T3–T4	0.16	0.40	0.022	0.01	0.02	0.699	0.53	0.29	0.043	0.01	0.02	0.663
Learning												
T1	−0.32	−0.32	0.024	–	–	–	−0.47	−0.10	0.337	–	–	–
T1–T2	−0.01	−0.04	0.916	−0.02	−0.17	0.198	0.54	0.44	0.008	−0.02	−0.10	0.112
T2–T4	−0.09	−0.37	0.200	0.01	0.06	0.512	−0.58	−0.54	0.041	0.01	0.05	0.360
Memory												
T1	−0.63	−0.41	0.002	–	–	–	−1.30	−0.10	0.000	–	–	–
T1–T2	0.25	0.18	0.288	−0.06	−0.06	0.501	0.62	0.44	0.144	−0.06	−0.05	0.488
T2–T3	0.06	0.10	0.673	0.05	0.13	0.142	0.23	0.15	0.300	0.05	0.10	0.143
T3–T4	−0.15	−0.50	0.260	−0.02	−0.11	0.074	−0.22	−0.30	0.222	−0.02	−0.10	0.080
Attention												
T1	−0.42	−0.38	0.001	–	–	–	−1.06	−0.21	0.002	–	–	–
T1–T2	0.50	0.54	0.191	0.10	0.16	0.096	1.75	0.43	0.001	0.06	0.07	0.304
T2–T3	−0.12	−0.26	0.903	−0.08	−0.24	0.003	−0.43	−0.19	0.225	−0.06	−0.14	0.047
T3–T4	−0.06	−0.21	0.684	0.03	0.17	0.070	0.63	0.56	0.003	0.02	0.11	0.040

The table presents results for all patients (i.e. total sample) and patients with CRP <10 mg/l (i.e. subsample). The time unit for change is months. Relations are given by unstandardised and standardised regression weights (*b* and β).

For the cognitive subdomains, we observed a statistically significant association between reduction of the CRP level and increase in verbal abilities for the T2–T4 interval and in attention for the T2–T3 interval (Table 4).

For patients with CRP <10 mg/l (*N*=191), the results remained unchanged regarding the association between changes in global cognitive performance (T2–T4), verbal abilities (T2–T4), and attention (T2–T3), and CRP level changes. However, we also found additional statistically significant associations in this sub-sample compared to the total sample (Table 4).

A separate model for the sub-sample of patients diagnosed within the schizophrenia spectrum showed that change in CRP level did not predict T2–T4 change in the global cognitive performance as was found in the total sample (*b*=−0.01, *p*=0.208). Two new statistically significant associations were found between change in CRP level and attention in the T1–T2 interval (*b*=0.12, *p*=0.045) and in the T3–T4 interval (*b*=0.03, *p*=0.026).

Finally, the models were adjusted for the covariates metabolic syndrome, smoking, being medication naïve, illicit drug use, and the educational level. The association between baseline CRP level and global cognitive performance for the whole follow-up remained essentially unchanged. Baseline CRP level and visuospatial performance in the T3–T4 interval was no longer statistically significant (*p*=0.120), whereas a statistically significant association was found between CRP baseline level and this subdomain in the T1–T2 interval (*b*=0.66, *p*=0.018). CRP level change and verbal abilities in the T2–T4 interval was no longer statistically significant associated (*p*=0.067), while such an association was found in the T1–T2 interval (*b*=−0.09, *p*=0.039). Additional statistically significant associations were found between CRP baseline level and attention in the T3–T4 interval (*b*=0.31, *p*<0.001), and CRP level change and attention in the T1–T2 interval (*b*=0.12, *p*=0.045), and in the T3–T4 (*b*=0.03, *p*=0.026), respectively.

A separate model for the sub-sample of patients diagnosed within the schizophrenia spectrum showed some differences. Change in CRP did not predict T2–T4 change in the global score as it did in the total sample (*b*=−0.01, *p*=0.208), whereas change in CRP was found to be associated with change in attention for the T1–T2 (*b*=0.12, *p*=0.045) and T3–T4 (*b*=0.03, *p*=0.026) intervals, respectively.

In the schizophrenia group, the inclusion of covariates showed that higher CRP baseline level was related to lower baseline level in global cognitive performance (*b*=−0.26, *p*=0.036). CRP baseline level was found to be associated with verbal abilities in the T1–T2 interval (*b*=0.64, *p*=0.016). In addition, CRP level change was found to be associated with verbal abilities in (*b*=−0.09, *p*=0.024) and learning in the T1–T2 interval (*b*=−0.04, *p*=0.009). The model with the attention outcome variable did not converge, even after increasing the number of iterations.

## Discussion

The main finding of the present study was that the global cognitive performance continued to improve from the initial phase (baseline to 4 weeks) of acute psychosis to the later phase (4 weeks to 6 months), and was predicted by the reduction of the CRP level as observed during the initial phase (baseline to 4 weeks) of the treatment. Similar associations were found for several of the cognitive subdomains. These findings might indicate a prolonged effect of inflammatory processes on cognition after an acute psychosis, stretching beyond the initial phase.

The stability of cognitive dysfunctions in patients with psychosis has been debated for decades. Most studies have suggested stability or decline in cognitive functioning (55–58). A study of first-episode schizophrenia spectrum disorders and controls with follow-up intervals of 1-year and 3-years showed that, although patients performed worse than controls at any given time, the cognitive performance of the patients improved in a similar way as the controls in all domains, except for verbal and visual memory, which showed greater improvement in controls (6). Another longitudinal study demonstrated improvement of general cognitive function, working memory, and verbal learning after 12 weeks, but these changes were mediated by improvements in both positive and negative symptoms (59).

There is substantial evidence that inflammatory processes are involved in the cognitive performance in psychosis (23,60,61). Oxidative stress and inflammation have been suggested to be associated with specific aspects of cognitive functioning in first-episode psychosis patients (62). Of particular interest, several studies have shown a link between CRP and cognitive function (25,29,37,63,64). CRP levels have been associated with cognitive impairment in patients with schizophrenia (25,36). In one study, abnormal CRP levels were correlated with poorer cognitive functioning of general intellectual ability, abstract reasoning, memory, working memory, semantic memory, learning abilities, attention, mental flexibility, and processing speed (25). In another, longitudinal study, CRP levels did not predict changes in cognitive performance (65), but this study included chronic phase patients with an average illness duration of 22.5 years, who are likely to have a lesser potential for inflammation related cognitive improvements.

Taken together, the present study supports a link between inflammation and cognitive performance in schizophrenia. Our study shows that early low-grade inflammation can predict cognitive improvement in later phases. Some studies have suggested that low-grade inflammation as indicated by elevated CRP levels is related to cerebral microstructural disintegration, involving frontal lobe executive functions (66). It has been suggested that increased levels of CRP can increase blood–brain barrier permeability, which might lead to inflammatory-related cognitive impairment (67). As neuroinflammation has been demonstrated to be associated with white matter pathology (27), any psychosis-related inflammation could, in principle, have a negative impact on brain tissue components of relevance to cognition, and the reversibility of such effects would likely include more slow regenerative processes.

There are some limitations to this study. First, attrition was substantial, but generally not related to any of the baseline characteristics except a higher PANSS negative subscale score and lower educational level in those patients tested only at baseline. As negative symptoms have been found to correlate with cognitive dysfunctions (12), those with the most pronounced cognitive difficulties may have dropped out during follow-up.

It is, however, difficult to predict how any selective dropout might have biased our results. Furthermore we used statistical methods that reduce the effect of missing data (68), and give improved statistical power and generalisability to the results, as all information in the data set is taken into consideration, rather than using the listwise deletion method that gives a net sample based on intact data in all variables under the assumption of missingness to be completely at random. However, the FIML method does not rule out the possibility of missingness to be non-random. The study included several models for different outcome variables in the total sample and in the schizophrenia sub-sample. In addition, several covariate variables were included in sensitivity analyses. This strategy has implication for statistical power, which has consequences particularly for small effects. Studies with larger samples are needed to increase the validity of our findings.

Being exploratory, we did not adjust for multiple testing. The high number of tests could theoretically result in statistical type I error. However, we find it very unlikely that such a high number of statistically significant findings could be the result of chance.

According to the naturalistic design, patients were included on the basis of the presence of active psychosis as determined by the PANSS, and not based on diagnosis. Hence all patients were psychotic and in need of antipsychotic medication. The lack of specific diagnosis for nine patients probably reflects that some patients may have dropped out or been discharged before the treating clinician were able to make a proper diagnostic evaluation. This is clearly a limitation, but the direction of any influence on our results is difficult to predict.

Moreover, CRP was the only inflammatory marker that was measured, at baseline and the first follow-up (T2). Further measurements during the follow-up period would have allowed for analyses of associations between potential later changes in the CRP level and cognitive performance. We are also aware of the limitation that other factors potentially confounding the relationship between CRP and cognition might exist that we have not accounted for, although many relevant factors were included in the analyses. Finally, the cognitive test battery at the T3 follow-up was more comprehensive but assessed the same cognitive domains as the test battery at T1, T2, and T4. We have no reason to suspect that this difference could have influenced the results.

We have shown that initial changes in the serum level of CRP in the acute phase of psychosis may predict cognitive function in later phases of the disease. These findings create an opportunity for future RCT research efforts to develop more individualised treatments with add-on anti-inflammatory agents (69). Some efforts have already been made in this field of research, but the results are mixed. A double-blind, randomised, placebo-controlled, add-on study with Celecoxib, a cyclo-oxygenase-2 inhibitor showed significant effect on the total PANSS score and the cognition factor of PANSS scale in patients with schizophrenia (70). N-acetyl cysteine significantly improved working memory compared to placebo in patients with psychosis, however, these preliminary data require replication (71). A double-blind, randomised study with minocycline treatment was associated with improvement in negative symptoms and executive functioning in early phase schizophrenia (72), whereas another study with add-on minocycline treatment showed improvement in negative symptoms, but not in cognition (73). Aspirin given as add-on treatment in another study reduced the total and positive PANSS score, but did not affect cognitive function (74). Finally, omega-3 fatty acid (ethyl eicosapentaenoic acid) add-on treatment did not affect cognition, positive or negative symptoms (75). Possible explanations for the equivocal findings might at least in part be related to small, unselected samples both with and without signs of an increased inflammatory status, with predominantly chronic phase psychosis, and with anti-inflammatory agents under investigation typically of low anti-inflammatory potency (76). Therefore, a more targeted approach might be to investigate more potent anti-inflammatory agents, for example, corticosteroids, in selected samples with signs of low-grade inflammation as determined by, for example, elevated CRP levels.

## References

[1] Kahn RS Keefe RS (2013) Schizophrenia is a cognitive illness: time for a change in focus. JAMA psychiatry 70, 1107–1112. 23925787 10.1001/jamapsychiatry.2013.155

[2] Green MF (1996) What are the functional consequences of neurocognitive deficits in schizophrenia? Am J Psychiatry 153, 321–330. 8610818 10.1176/ajp.153.3.321

[3] Howes OD Murray RM (2014) Schizophrenia: an integrated sociodevelopmental-cognitive model. Lancet (London, England) 383, 1677–1687. 10.1016/S0140-6736(13)62036-X PMC4127444 24315522

[4] Kroken RA, Loberg EM, Dronen T, Gruner R, Hugdahl K, Kompus K, Skrede S Johnsen E (2014) A critical review of pro-cognitive drug targets in psychosis: convergence on myelination and inflammation. Front Psychiatry 5, 11. 24550848 10.3389/fpsyt.2014.00011 PMC3912739

[5] Heaton RK, Gladsjo JA, Palmer BW, Kuck J, Marcotte TD Jeste DV (2001) Stability and course of neuropsychological deficits in schizophrenia. Arch Gen Psychiatry 58, 24–32. 11146755 10.1001/archpsyc.58.1.24

[6] Rodriguez-Sanchez JM, Ayesa-Arriola R, Perez-Iglesias R, Perianez JA, Martinez-Garcia O, Gomez-Ruiz E, Tabares-Seisdedos R Crespo-Facorro B (2013) Course of cognitive deficits in first episode of non-affective psychosis: a 3-year follow-up study. Schizophr Res 150, 121–128. 23899999 10.1016/j.schres.2013.06.042

[7] Ito S, Nemoto T, Tsujino N, Ohmuro N, Matsumoto K, Matsuoka H, Tanaka K, Nishiyama S, Brunel L, Suzuki M, Kinoshita H, Ozawa H, Fujita H, Shimodera S, Kishimoto T, Matsumoto K, Hasegawa T Mizuno M (2015) Differential impacts of duration of untreated psychosis (DUP) on cognitive function in first-episode schizophrenia according to mode of onset. Eur Psychiatry 30, 995–1001. 26497470 10.1016/j.eurpsy.2015.08.004

[8] Johnsen E, Jorgensen HA, Kroken RA Loberg EM (2013) Neurocognitive effectiveness of quetiapine, olanzapine, risperidone, and ziprasidone: a pragmatic, randomized trial. Eur Psychiatry 28, 174–184. 22153730 10.1016/j.eurpsy.2011.10.003

[9] Rapado-Castro M, Dodd S, Bush AI, Malhi GS, Skvarc DR, On ZX, Berk M Dean OM (2016) Cognitive effects of adjunctive N-acetyl cysteine in psychosis. Psychol Med 47, 1–11. 27894373 10.1017/S0033291716002932

[10] Martinez-Cengotitabengoa M, Mico JA, Arango C, Castro-Fornieles J, Graell M, Paya B, Leza JC, Zorrilla I, Parellada M, Lopez MP, Baeza I, Moreno C, Rapado-Castro M Gonzalez-Pinto A (2014) Basal low antioxidant capacity correlates with cognitive deficits in early onset psychosis. A 2-year follow-up study. Schizophr Res 156, 23–29. 24768133 10.1016/j.schres.2014.03.025

[11] Johnsen E, Fathian F, Kroken RA, Steen VM, Jorgensen HA, Gjestad R Loberg EM (2016) The serum level of C-reactive protein (CRP) is associated with cognitive performance in acute phase psychosis. BMC Psychiatry 16, 60. 26973142 10.1186/s12888-016-0769-x PMC4790054

[12] Anda L, Bronnick KS, Johnsen E, Kroken RA, Jorgensen H Loberg EM (2016) The course of neurocognitive changes in acute psychosis: relation to symptomatic improvement. PloS One 11, e0167390. 27977720 10.1371/journal.pone.0167390 PMC5157988

[13] Lee WH Lee WK (2017) Cognitive rehabilitation for patients with schizophrenia in Korea. Asian J Psychiatr 25, 109–117. 28262129 10.1016/j.ajp.2016.10.010

[14] Best MW Bowie CR (2017) A review of cognitive remediation approaches for schizophrenia: from top-down to bottom-up, brain training to psychotherapy. Expert Rev Neurother 17, 713–723. 28511562 10.1080/14737175.2017.1331128

[15] Turkington D Morrison AP (2012) Cognitive therapy for negative symptoms of schizophrenia. Arch Gen Psychiatry 69, 119–120. 21969421 10.1001/archgenpsychiatry.2011.141

[16] Davidson M, Galderisi S, Weiser M, Werbeloff N, Fleischhacker WW, Keefe RS, Boter H, Keet IP, Prelipceanu D, Rybakowski JK, Libiger J, Hummer M, Dollfus S, Lopez-Ibor JJ, Hranov LG, Gaebel W, Peuskens J, Lindefors N, Riecher-Rossler A Kahn RS (2009) Cognitive effects of antipsychotic drugs in first-episode schizophrenia and schizophreniform disorder: a randomized, open-label clinical trial (EUFEST). Am J Psychiatry 166, 675–682. 19369319 10.1176/appi.ajp.2008.08060806

[17] Nielsen RE, Levander S, Kjaersdam Telleus G, Jensen SO, Ostergaard Christensen T Leucht S (2015) Second-generation antipsychotic effect on cognition in patients with schizophrenia--a meta-analysis of randomized clinical trials. Acta Psychiatr Scand 131, 185–196. 25597383 10.1111/acps.12374

[18] Dickerson F, Stallings C, Origoni A, Vaughan C, Khushalani S Yolken R (2013) Elevated C-reactive protein and cognitive deficits in individuals with bipolar disorder. J Affect Disord 150, 456–459. 23684514 10.1016/j.jad.2013.04.039

[19] Khandaker GM Dantzer R (2016) Is there a role for immune-to-brain communication in schizophrenia? Psychopharmacology 233, 1559–1573. 26037944 10.1007/s00213-015-3975-1 PMC4671307

[20] Fischer EK Drago A (2017) A molecular pathway analysis stresses the role of inflammation and oxidative stress towards cognition in schizophrenia. J Neural Trans 124, 765–774. 10.1007/s00702-017-1730-y 28477285

[21] Khandaker GM, Cousins L, Deakin J, Lennox BR, Yolken R Jones PB (2015) Inflammation and immunity in schizophrenia: implications for pathophysiology and treatment. Lancet Psychiatry 2, 258–270. 26359903 10.1016/S2215-0366(14)00122-9 PMC4595998

[22] Stefansson H, Ophoff RA Steinberg S (2009) Common variants conferring risk of schizophrenia. Nature 460. 10.1038/nature08186 PMC3077530 19571808

[23] Miller BJ Goldsmith DR (2017) Towards an immunophenotype of schizophrenia: progress, potential mechanisms, and future directions. Neuropsychopharmacology 42, 299–317. 27654215 10.1038/npp.2016.211 PMC5143505

[24] Ribeiro-Santos A, Lucio Teixeira A Salgado JV (2014) Evidence for an immune role on cognition in schizophrenia: a systematic review. Curr Neuropharmacol 12, 273–280. 24851091 10.2174/1570159X1203140511160832 PMC4023457

[25] Bulzacka E, Boyer L, Schurhoff F, Godin O, Berna F, Brunel L, Andrianarisoa M, Aouizerate B, Capdevielle D, Chereau-Boudet I, Chesnoy-Servanin G, Danion JM, Dubertret C, Dubreucq J, Faget C, Gabayet F, Le Gloahec T, Llorca PM, Mallet J, Misdrahi D, Rey R, Richieri R, Passerieux C, Roux P, Yazbek H, Leboyer M Fond G (2016) Chronic peripheral inflammation is associated with cognitive impairment in schizophrenia: results from the multicentric FACE-SZ dataset. Schizophr Bull 42, 1290–1302. 27143795 10.1093/schbul/sbw029 PMC4988740

[26] Kahn RS Sommer IE (2015) The neurobiology and treatment of first-episode schizophrenia. Mol Psychiatry 20, 84–97. 25048005 10.1038/mp.2014.66 PMC4320288

[27] Najjar S Pearlman DM (2015) Neuroinflammation and white matter pathology in schizophrenia: systematic review. Schizophr Res 161, 102–112. 24948485 10.1016/j.schres.2014.04.041

[28] Deodhar SD (1989) C-reactive protein: the best laboratory indicator available for monitoring disease activity. Cleve Clin J Med 56, 126–130. 2659202 10.3949/ccjm.56.2.126

[29] Horsdal HT, Kohler-Forsberg O, Benros ME Gasse C (2017) C-reactive protein and white blood cell levels in schizophrenia, bipolar disorders and depression - associations with mortality and psychiatric outcomes: a population-based study. Eur Psychiatry 44, 164–172. 28645055 10.1016/j.eurpsy.2017.04.012

[30] Fan X, Pristach C, Liu EY, Freudenreich O, Henderson DC Goff DC (2007) Elevated serum levels of C-reactive protein are associated with more severe psychopathology in a subgroup of patients with schizophrenia. Psychiatr Res 149, 267–271. 10.1016/j.psychres.2006.07.011 17112596

[31] Dickerson F, Stallings C, Origoni A, Vaughan C, Khushalani S, Yang S Yolken R (2013) C-reactive protein is elevated in schizophrenia. Schizophr Res 143, 198–202. 23218564 10.1016/j.schres.2012.10.041

[32] Inoshita M, Numata S, Tajima A, Kinoshita M, Umehara H, Nakataki M, Ikeda M, Maruyama S, Yamamori H, Kanazawa T, Shimodera S, Hashimoto R, Imoto I, Yoneda H, Iwata N Ohmori T (2016) A significant causal association between C-reactive protein levels and schizophrenia. Sci Rep 6, 26105. 27193331 10.1038/srep26105 PMC4872134

[33] Fernandes BS, Steiner J, Bernstein HG, Dodd S, Pasco JA, Dean OM, Nardin P, Goncalves CA Berk M (2016) C-reactive protein is increased in schizophrenia but is not altered by antipsychotics: meta-analysis and implications. Mol Psychiatry 21, 554–564. 26169974 10.1038/mp.2015.87

[34] Wium-Andersen MK, Orsted DD Nordestgaard BG (2014) Elevated C-reactive protein associated with late- and very-late-onset schizophrenia in the general population: a prospective study. Schizophr Bulletin 40, 1117–1127. 10.1093/schbul/sbt120 PMC4133657 23996346

[35] Miller BJ, Culpepper N Rapaport MH (2014) C-reactive protein levels in schizophrenia: a review and meta-analysis. Clin Schizophr Relat Psychoses 7, 223–230. 23428789

[36] Dickerson F, Stallings C, Origoni A, Boronow J Yolken R (2007) C-reactive protein is associated with the severity of cognitive impairment but not of psychiatric symptoms in individuals with schizophrenia. Schizophr Res 93, 261–265. 17490859 10.1016/j.schres.2007.03.022

[37] Dickerson F, Stallings C, Origoni A, Vaughan C, Khushalani S Yolken R (2012) Additive effects of elevated C-reactive protein and exposure to Herpes Simplex Virus type 1 on cognitive impairment in individuals with schizophrenia. Schizophr Res 134, 83–88. 22048011 10.1016/j.schres.2011.10.003

[38] Johnsen E, Kroken RA, Wentzel-Larsen T Jorgensen HA (2010) Effectiveness of second-generation antipsychotics: a naturalistic, randomized comparison of olanzapine, quetiapine, risperidone, and ziprasidone. BMC Psychiatry 10, 26. 20334680 10.1186/1471-244X-10-26 PMC2851682

[39] Kay SR, Fiszbein A Opler LA (1987) The positive and negative syndrome scale (PANSS) for schizophrenia. Schizophr Bull 13, 261–276. 3616518 10.1093/schbul/13.2.261

[40] Robinson DG, Woerner MG, Alvir JM, Geisler S, Koreen A, Sheitman B, Chakos M, Mayerhoff D, Bilder R, Goldman R Lieberman JA (1999) Predictors of treatment response from a first episode of schizophrenia or schizoaffective disorder. Am J Psychiatry 156, 544–549. 10200732 10.1176/ajp.156.4.544

[41] Addington D, Addington J Schissel B (1990) A depression rating scale for schizophrenics. Schizophr Res 3, 247–251. 2278986 10.1016/0920-9964(90)90005-r

[42] Drake RE, Rosenberg SD Mueser KT (1996) Assessing substance use disorder in persons with severe mental illness. New Dir Ment Health Serv 70, 3–17. 10.1002/yd.23319960203 8754227

[43] Guy W (1976) ECDEU Assessment Manual for Psychopharmacology. Rockville,MD: Department of Health, Education, and Welfare.

[44] Pedersen G Karterud S (2012) The symptom and function dimensions of the Global Assessment of Functioning (GAF) scale. Compr Psychiatry 53, 292–298. 21632038 10.1016/j.comppsych.2011.04.007

[45] Beglinger LJ, Gaydos B, Tangphao-Daniels O, Duff K, Kareken DA, Crawford J, Fastenau PS Siemers ER (2005) Practice effects and the use of alternate forms in serial neuropsychological testing. Arch Clin Neuropsychol 20, 517–529. 15896564 10.1016/j.acn.2004.12.003

[46] Gold JM, Queern C, Iannone VN Buchanan RW (1999) Repeatable battery for the assessment of neuropsychological status as a screening test in schizophrenia I: sensitivity, reliability, and validity. Am J Psychiatry 156, 1944–1950. 10588409 10.1176/ajp.156.12.1944

[47] Wilk CM, Gold JM, Bartko JJ, Dickerson F, Fenton WS, Knable M, Randolph C Buchanan RW (2002) Test-retest stability of the repeatable battery for the assessment of neuropsychological status in schizophrenia. Am J Psychiatry 159, 838–844. 11986139 10.1176/appi.ajp.159.5.838

[48] Goldberg TE, Keefe RS, Goldman RS, Robinson DG Harvey PD (2010) Circumstances under which practice does not make perfect: a review of the practice effect literature in schizophrenia and its relevance to clinical treatment studies. Neuropsychopharmacology 35, 1053–1062. 20090669 10.1038/npp.2009.211 PMC3055399

[49] Goldberg TE, Goldman RS, Burdick KE, Malhotra AK, Lencz T, Patel RC, Woerner MG, Schooler NR, Kane JM Robinson DG Cognitive improvement after treatment with second-generation antipsychotic medications in first-episode schizophrenia: is it a practice effect? Arch Gen Psychiatry 2007;64, 1115–1122. 17909123 10.1001/archpsyc.64.10.1115

[50] Loberg EM, Langeland M Jorgensen HA (2006) Reliability and validity of a Norwegian translation of the repeatable battery for the assessment of neuropsychological status in patients with psychosis. Nevropsykologi 9, 36.

[51] Muthén LK Muthén BO (2017) Mplus 8. Los Angeles, CA: Muthén & Muthén.

[52] Wang J Wang X (2012) Structural Equation Modeling: Applications Using Mplus Wiley Series in Probability and Statistics. West Sussex: Wiley, A John Wiley & Sons Ltd.

[53] Newsom JT (2015) Longitudinal Structural Equation Modeling. New York, NY: Routledge, Taylor & Francis Group.

[54] Kline RB (2010) Principles and Practice of Structural Equation Modeling, 3rd edn New York, NY: The Guiford Press.

[55] Bilder RM, Lipschutz-Broch L, Reiter G, Geisler SH, Mayerhoff DI Lieberman JA (1992) Intellectual deficits in first-episode schizophrenia: evidence for progressive deterioration. Schizophr Bull 18, 437–448. 1411331 10.1093/schbul/18.3.437

[56] Nopoulos P, Flashman L, Flaum M, Arndt S Andreasen N (1994) Stability of cognitive functioning early in the course of schizophrenia. Schizophr Res 14, 29–37. 7893619 10.1016/0920-9964(94)90006-x

[57] Liberman RP Green MF (1992) Whither cognitive-behavioral therapy for schizophrenia? Schizophr Bull 18, 27–35. 1553496 10.1093/schbul/18.1.27

[58] Tyson PJ, Laws KR, Roberts KH Mortimer AM (2004) Stability of set-shifting and planning abilities in patients with schizophrenia. Psychiatr Res 129, 229–239. 10.1016/j.psychres.2004.09.007 15661316

[59] Trampush JW, Lencz T, DeRosse P, John M, Gallego JA, Petrides G, Hassoun Y, Zhang JP, Addington J, Kellner CH, Tohen M, Burdick KE, Goldberg TE, Kane JM, Robinson DG Malhotra AK (2015) Relationship of cognition to clinical response in first-episode schizophrenia spectrum disorders. Schizophr Bull 41, 1237–1247. 26409223 10.1093/schbul/sbv120 PMC4601719

[60] Mondelli V, Pariante CM, Navari S, Aas M, D’Albenzio A Di Forti M, Handley R, Hepgul N, Marques TR, Taylor H, Papadopoulos AS, Aitchison KJ, Murray RM Dazzan P (2010) Higher cortisol levels are associated with smaller left hippocampal volume in first-episode psychosis. Schizophr Res 119, 75–78. 20071148 10.1016/j.schres.2009.12.021 PMC3513409

[61] Buckley PF, Pillai A, Evans D, Stirewalt E Mahadik S (2007) Brain derived neurotropic factor in first-episode psychosis. Schizophr Res 91, 1–5. 17306505 10.1016/j.schres.2006.12.026 PMC1933504

[62] Martinez-Cengotitabengoa M, Mac-Dowell KS, Leza JC, Mico JA, Fernandez M Echevarria E, Sanjuan J, Elorza J Gonzalez-Pinto A (2012) Cognitive impairment is related to oxidative stress and chemokine levels in first psychotic episodes. Schizophr Res 137, 66–72. 22445462 10.1016/j.schres.2012.03.004

[63] Micoulaud-Franchi JA, Faugere M, Boyer L, Fond G, Richieri R, Faget C, Cermolacce M, Philip P, Vion-Dury J Lancon C (2015) Elevated C-reactive protein is associated with sensory gating deficit in schizophrenia. Schizophr Res 165, 94–96. 25864954 10.1016/j.schres.2015.03.018

[64] Misiak B, Stanczykiewicz B, Kotowicz K, Rybakowski JK, Samochowiec J Frydecka D (2018) Cytokines and C-reactive protein alterations with respect to cognitive impairment in schizophrenia and bipolar disorder: a systematic review. Schizophr Res 192, 16–29. 28416092 10.1016/j.schres.2017.04.015

[65] Dickerson F, Schroeder J, Stallings C, Origoni A, Katsafanas E Schwienfurth LA, Savage CL, Khushalani S Yolken R (2014) A longitudinal study of cognitive functioning in schizophrenia: clinical and biological predictors. Schizophr Res 156, 248–253. 24827555 10.1016/j.schres.2014.04.019

[66] Wersching H, Duning T, Lohmann H, Mohammadi S, Stehling C, Fobker M, Conty M, Minnerup J, Ringelstein EB, Berger K, Deppe M Knecht S (2010) Serum C-reactive protein is linked to cerebral microstructural integrity and cognitive function. Neurology 74, 1022–1029. 20350977 10.1212/WNL.0b013e3181d7b45b

[67] Hsuchou H, Kastin AJ, Mishra PK Pan W (2012) C-reactive protein increases BBB permeability: implications for obesity and neuroinflammation. Cell Physiol Biochem 30, 1109–1119. 23018453 10.1159/000343302 PMC4099000

[68] Bollen KA (2002) Latent variables in psychology and the social sciences. Annu Rev Psychol 53, 605–634. 11752498 10.1146/annurev.psych.53.100901.135239

[69] Bumb JM, Enning F Leweke FM (2015) Drug repurposing and emerging adjunctive treatments for schizophrenia. Expert Opin Pharmacother 16, 1049–1067. 25866122 10.1517/14656566.2015.1032248

[70] Muller N, Riedel M, Schwarz MJ Engel RR (2005) Clinical effects of COX-2 inhibitors on cognition in schizophrenia. Eur Arch Psychiatry Clin Neurosci 255, 149–151. 15549344 10.1007/s00406-004-0548-4

[71] Rapado-Castro M, Dodd S, Bush AI, Malhi GS, Skvarc DR, On ZX, Berk M Dean OM (2017) Cognitive effects of adjunctive N-acetyl cysteine in psychosis. Psychol Med 47, 866–876. 27894373 10.1017/S0033291716002932

[72] Levkovitz Y, Mendlovich S, Riwkes S, Braw Y, Levkovitch-Verbin H, Gal G, Fennig S, Treves I Kron S (2010) A double-blind, randomized study of minocycline for the treatment of negative and cognitive symptoms in early-phase schizophrenia. J Clin Psychiatry 71, 138–149. 19895780 10.4088/JCP.08m04666yel

[73] Chaudhry IB, Hallak J, Husain N, Minhas F, Stirling J, Richardson P, Dursun S, Dunn G Deakin B (2012) Minocycline benefits negative symptoms in early schizophrenia: a randomised double-blind placebo-controlled clinical trial in patients on standard treatment. J Psychopharmacol 26, 1185–1193. 22526685 10.1177/0269881112444941

[74] Laan W, Grobbee DE, Selten JP, Heijnen CJ, Kahn RS Burger H (2010) Adjuvant aspirin therapy reduces symptoms of schizophrenia spectrum disorders: results from a randomized, double-blind, placebo-controlled trial. J Clin Psychiatry 71, 520–527. 20492850 10.4088/JCP.09m05117yel

[75] Fenton WS, Dickerson F, Boronow J, Hibbeln JR Knable M (2001) A placebo-controlled trial of omega-3 fatty acid (ethyl eicosapentaenoic acid) supplementation for residual symptoms and cognitive impairment in schizophrenia. Am J Psychiatry 158, 2071–2074. 11729030 10.1176/appi.ajp.158.12.2071

[76] Sommer IE, van Westrhenen R, Begemann MJ, de Witte LD, Leucht S Kahn RS (2014) Efficacy of anti-inflammatory agents to improve symptoms in patients with schizophrenia: an update. Schizophr Bull 40, 181–191. 24106335 10.1093/schbul/sbt139 PMC3885306

