# Supplementary material for: Associations between C-reactive protein levels and cognition during the first 6 months after acute psychosis
Source: Acta Neuropsychiatr. 2018 Nov 5;31(1):36–45. doi: 10.1017/neu.2018.25 (PMC6468237; doi:10.1017/neu.2018.25)
Supplement: Supplementary file 1 [file S092427081800025Xsup001.docx]

**Supplementary table 1.** **Goodness of fit measures for level and change models of cognitive tests.**

|  |  | **χ^2^** |  | **df** | **p** |  | **CFI** | **TLI** |  | **RMSEA** | | |  |
| --- | --- | --- | --- | --- | --- | --- | --- | --- | --- | --- | --- | --- | --- |
| **Scale** ^a^ |  |  |  |  |  |  |  |  |  | **estimate** | **C.I.** | **P_close fit_** |  |
| **Global performance** |  | 2.36 |  | 3 | .501 |  | 1.00 | 1.01 |  | .000 | .000-.115 | .67 |  |
| **Verbal abilities** |  | 4.82 |  | 3 | .186 |  | 0.96 | 0.92 |  | .058 | .000-.149 | .35 |  |
| **Learning** |  | 2.57 |  | 3 | .462 |  | 1.00 | 1.01 |  | .000 | .000-.118 | .64 |  |

**Notes:**

^a^ For Visuospatial abilities, Memory, and Attention, saturated models had to be estimated

(df = 0). Thus, no model fit is estimated for these models.
